# MicroRNAs and the regulation of intestinal homeostasis

**DOI:** 10.3389/fgene.2014.00347

**Published:** 2014-10-01

**Authors:** Marah C. Runtsch, June L. Round, Ryan M. O’Connell

**Affiliations:** Department of Pathology, University of UtahSalt Lake City, UT, USA

**Keywords:** microRNAs, intestine, microbiota, immune system, homeostasis, host-commensal

## Abstract

The mammalian intestinal tract is a unique site in which a large portion of our immune system and the 10^14^ commensal organisms that make up the microbiota reside in intimate contact with each other. Despite the potential for inflammatory immune responses, this complex interface contains host immune cells and epithelial cells interacting with the microbiota in a manner that promotes symbiosis. Due to the complexity of the cell types and microorganisms involved, this process requires elaborate regulatory mechanisms to ensure mutualism and prevent disease. While many studies have described critical roles for protein regulators of intestinal homeostasis, recent reports indicate that non-coding RNAs are also major contributors to optimal host-commensal interactions. In particular, there is emerging evidence that microRNAs (miRNAs) have evolved to fine tune host gene expression networks and signaling pathways that modulate cellular physiology in the intestinal tract. Here, we review our present knowledge of the influence miRNAs have on both immune and epithelial cell biology in the mammalian intestines and the impact this has on the microbiota. We also discuss a need for further studies to decipher the functions of specific miRNAs within the gut to better understand cellular mechanisms that promote intestinal homeostasis and to identify potential molecular targets underlying diseases such as inflammatory bowel disease and colorectal cancer.

## INTRODUCTION

A major goal of the biomedical research community is to understand mechanisms that regulate the gut immune system in a manner that maintains homeostasis, despite the presence of trillions of bacteria and other microorganisms that reside on and within the host. Gut homeostasis is defined by a proper balance of pro-inflammatory responses against harmful and/or invading microbes while tolerating non-invasive and beneficial microbes (Hooper and Macpherson, 2010). During this state, healthy proportions of commensals, tolerant immune cells, and pro-inflammatory host cells interact closely with one another. However, if this balance is disrupted, either by changes to the composition of the gut microbiota or by alterations to the host response, diseases can emerge (Round and Mazmanian, 2009).

**FIGURE 1:**
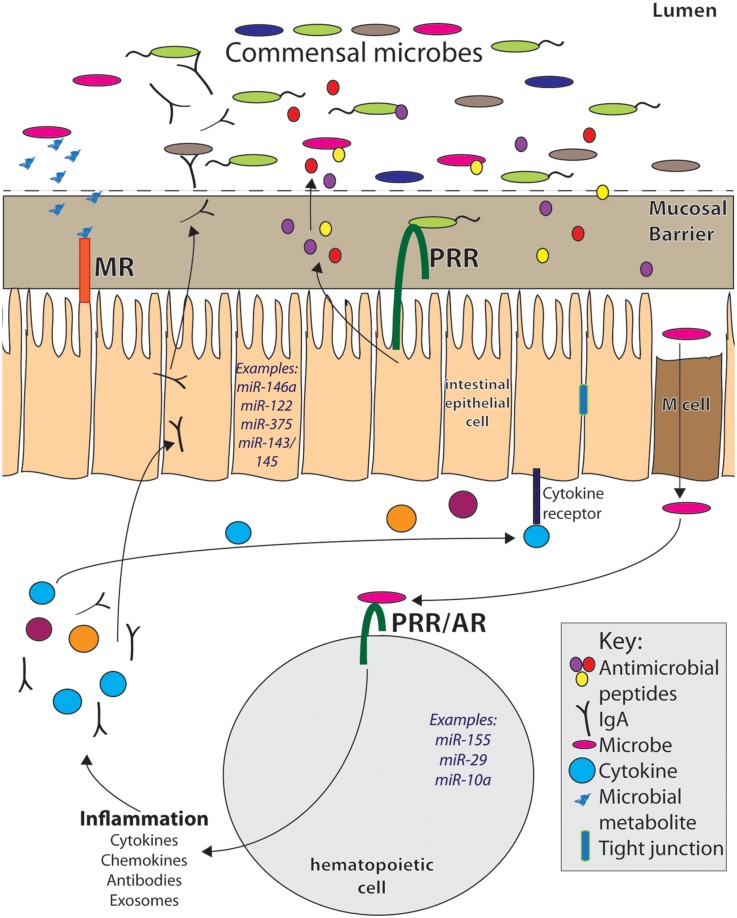
**MicroRNAs play important roles within the complex intestinal immune system.** They can be expressed within hematopoietic cells in response to inflammatory signals from pattern recognition receptors (PRR) and antigen receptors (AR). In this way, miRNAs can regulate immune responses, including secretion of cytokines, chemokines, and antibodies, all of which affect intestinal homeostasis. Within intestinal epithelial cells (IECs) and other non-hematopoietic intestinal cells, miRNAs are expressed in and regulate pathways involved in secretion of antimicrobial peptides, cell renewal, and barrier permeability, among others. They also may mediate host responses to microbial metabolites, which signal to host cells via metabolite receptors (MR). Many immune signals that induce miRNAs and immune responses within the gut come from the microbiota. Altered commensal populations may mediate different miRNA responses and functions within the intestinal tract.

In order to maintain homeostasis, the host intestinal mucosa contains distinct regions consisting of various cell types necessary to respond to antigens in an appropriate manner (Hooper and Macpherson, 2010; **Figure 1**). These regions include: the outer and inner mucus layers, in which invading microbes become trapped to prevent spread of infection (Johansson et al., 2011); the epithelial layer, in which physical and chemical barriers are formed to prevent dissemination of bacteria into underlying tissues (Goto and Ivanov, 2013; Peterson and Artis, 2014); the lamina propria (LP), a leukocyte-rich region that lies underneath the epithelium, housing cells that respond to microbial signals (Duerkop et al., 2009); and other immune cell-containing gut-associated lymphoid tissues (GALT) such as Peyer’s Patches and mesenteric lymph nodes. Immune cell-rich gut tissues contain a diverse set of cell types, both hematopoietic and non-hematopoietic-derived, that play a role in maintaining healthy interactions with the resident microbiota. Some of these cell types are unique to the intestines and many have distinct functions within the GI tract. Several of these gut cells have been demonstrated to be required for providing both defense against pathogens and tolerance to commensals in order to prevent disease.

The gut microbiota itself heavily influences proper development and function of the intestinal immune system (Round and Mazmanian, 2009; Hooper et al., 2012; Ivanov and Honda, 2012). Indeed, germ-free mice display developmental defects within GALT and intestinal epithelial cells (IECs). One mechanism by which the microbiota can influence intestinal immune cells is via surface and cytoplasmic receptors called pattern recognition receptors (PRRs) that recognize conserved microbial motifs on both pathogens and commensals. PRR detection of microbial products initiates a variety of immune responses and developmental pathways. One class of PRRs is the toll-like receptors (TLRs), which signal primarily through the adaptor protein MyD88 to activate master transcription factors such as NF-κB. In some cases, gut microbial recognition by the host leads to proinflammatory responses resulting in increased gut inflammation (Ivanov et al., 2009). In other situations, activation of cell surface receptors by commensal products induces tolerogenic responses (Round et al., 2011; Smith et al., 2013). Additionally, recognition of commensal and food molecules by PRRs is essential for the maintenance of intestinal homeostasis (Rakoff-Nahoum et al., 2004; Abreu, 2010). This demonstrates the importance of host-commensal interactions in proper immune function and overall host physiology. While PRRs are but one mechanism thought to influence homeostasis, many of the molecular events that mediate crosstalk between host cells and microbiota remain enigmatic.

MicroRNAs (miRNAs) have recently emerged as important mediators of immune development and responses. miRNAs are short (21–25 nucleotide), non-coding RNA molecules that are most commonly transcribed by RNA polymerase II and processed by proteins such as Drosha and Dicer (Winter et al., 2009). In their mature, RNA-induced silencing complex (RISC)-bound form, miRNAs bind to and downregulate expression of target mRNAs by degradation and/or blocking of translation. miRNAs have been demonstrated to regulate immune responses by modulating gene expression of immune-related genes. In this way, they modulate the balance between effective inflammatory responses to foreign entities and proper resolution of inflammation to prevent tissue damage. More than 100 miRNAs are expressed within leukocytes (O’Connell et al., 2010), and many of these miRNAs have multiple targets within immune-related pathways, consistent with an important role in the immune system (Baltimore et al., 2008; Lindsay, 2008; O’Connell et al., 2010, 2012; Chen et al., 2013). Some of the most well-studied miRNAs in the immune system include miR-155, miR-146a, miRs-17∼92, and miR-181a (Baltimore et al., 2008). Many of these miRNAs regulate key signaling pathways such as Jak/Stat, TLR/MyD88, NF-κB, and Akt, which are essential for immune responses (O’Connell et al., 2012). Some miRNAs, such as miR-155, target negative regulators of the immune response to promote inflammation. Conversely, others, like miR-146a, target positive regulators of immunity to promote tolerance and resolution of responses. Despite the large amount of work done over the past decade, not all targets and functions of miRNAs in the immune system, including well-studied miRNAs, have been worked out. Furthermore, even less is known about miRNAs within the intestinal immune system.

microRNAs modulate expression of genes involved in microbial recognition and downstream immune activity, and thus may play an important role within the intestinal immune system during its interactions with the gut microbiota. Their expression in various intestinal cell types, including hematopoietic-derived leukocytes, IECs, and other specialized gut cell types points to a role in mediating homeostasis with commensal microbes and overall intestinal health (**Figure 1**). These non-coding RNAs function as rheostats to the immune response as opposed to binary switches as they act to adjust the magnitude of gene expression. miRNAs also work in feedback loops, ensuring that the immune system does not produce inappropriately strong responses while also promoting protective immunity when needed. In this way, miRNAs themselves can be considered mediators of homeostasis within the immune system as they act to buffer inflammation. Immune processes within the GI tract are coordinated differently when compared to other sites in the body as a result of regulatory mechanisms required to handle the constant exposure to microbes. Intestinal homeostasis is sensitive to even small disruptions, and therefore miRNAs make excellent candidate molecules for fine-tuning responses at this sensitive site. Multiple studies are beginning to demonstrate the importance of miRNAs within the intestine and identifying how the microbiota influences miRNA expression and function within the GI tract; some of this work will be reviewed here (**Table 1**).

**Table 1:** Selected summary of studied miRNAs and their roles in the intestinal immune system, reviewed in this paper.

miRNA	Intestinal role or effect	Compartment and/or cell types involved	miRNA target(s)	Reference
miR-155	Induced by TGFβ, decreased IL-2 and IFNγ expression in lamina propria	LP T cells	InducibleT cell kinase (ITK)	Das et al. (2013)
miR-29	Decreased IL-23/Th17 gut responses, protected from colitis	Dendritic cells	IL-12p40	Brain et al. (2013)
miR-10a	Maintained Treg lineage and prevented plasticity to other T cell subsets	Peyer’s patch T cells	Bcl6	Takahashi et al. (2012)
miR-146a	Reduced inflammation during intestinal ischemia reperfusion injury	Intestinal epithelial cells	IRAKI	Chassin et al. (2012)
miR-122	Increased intestinal permeability when induced with TNFa	Intestinal epithelial cells	Occludin	Ye et al. (2011)
miR-124	Associated with protection from pediatric UC	Human colonocytes	Stat3	Koukos et al. (2013)
miR-21	Overexpressed in intestinal disease, knockout mice protected from colitis	Whole murine colon	Unknown	Shi et al. (2013)
miR-143/ mlR-145	Intestinal epithelial regeneration following tissue injury (DSS)	Mesenchymal cells	Igfbp5	Chivukula et al. (2014)

## miRNAs IN HEMATOPOIETIC-DERIVED GUT IMMUNE CELLS

The function of miRNAs within hematopoietic-derived immune cells has been well-studied, and it is now clear that miRNAs are deeply integrated into the molecular networks that govern mammalian immune responses (Lindsay, 2008; O’Connell et al., 2010, 2012; Chen et al., 2013). However, relatively few studies have examined the functions of miRNAs in leukocytes within the GI tract, where unique subsets of immune cells are known to reside. Recent investigations indicate that miRNAs also play important roles in hematopoietic cells within the intestines. For example, miR-155 was found to be required for protection during mucosal infection by the intestinal pathogen *Citrobacter rodentium* (Clare et al., 2013). miR-155-deficient mice displayed a higher pathogen burden within the gut, a delayed ability to clear the bacteria, and a worsened colitis phenotype as a result of infection. Interestingly, this phenotype was attributed to a defective intestinal humoral response due to a loss of miR-155 in B cells. This study demonstrates that miR-155 regulates gut B cells in a manner similar to its functions in B cells found in other microenvironments (Thai et al., 2007; Vigorito et al., 2007); however, a different study displayed a unique role for miR-155 in the gut. Upon exposure to TGFβ, miR-155 was found to be significantly upregulated in LP T cells, contrasting with only a modest induction of miR-155 when peripheral T cells were treated with TGFβ (Das et al., 2013). Additionally, while miR-155 is known to promote inflammatory responses in many contexts, Das et al. (2013) observed immune-inhibitory effects of miR-155 in LP T cells, as evidenced by their intrinsic downregulation of both IL-2 and IFNγ expression upon induction of miR-155 by TGFβ. Importantly, gain and loss of function approaches revealed that miR-155 is indeed a repressor of these cytokines in LP T cells and identified a prominent target of miR-155 in LP T cells as inducible T-cell kinase (ITK), which normally promotes development and effector function of T cells. This work provides evidence that miR-155 may function uniquely within intestinal LP T cells, in contrast to its well-studied roles in T cells found at other anatomical locations.

A unique role for miR-29 was recently demonstrated in dendritic cells of the intestine. In this study, the intracellular pattern recognition receptor NOD2 induced expression of miR-29 in dendritic cells (Brain et al., 2013). miR-29 was found to directly target IL-12p40, which led to the downregulation of IL-23 and Th17 responses in the gut. Consequently, mice lacking miR-29 displayed worsened microbial-dependent colitis compared to WT controls. These data characterized a gut-specific role for miR-29, which had previously been shown to target genes involved in proliferation, differentiation, and fighting bacterial infections (Ma et al., 2011). These findings provide additional evidence that miRNA functions can be unique within the intestinal microenvironment compared to their functions in peripheral sites, and this may also involve their expression and function in distinct cell types.

miR-10a, a miRNA largely involved in embryonic development (Lund, 2010), has also been shown to play a distinct role within in the gut. In Peyer’s Patches, which are located throughout the small intestine, miR-10a was highly expressed within regulatory T cells (Tregs; Takahashi et al., 2012). Expression of this miRNA within inducible Tregs in the Peyer’s Patch constrained their plasticity and prevented conversion into T follicular helper (Tfh) cells. Takahashi et al. (2012) demonstrated that miR-10a maintained the Treg lineage within the GALT by targeting and downregulating the Tfh master regulator gene, Bcl6. The biological significance of miR-10a function within Peyer’s Patch Treg cells appears to be the maintainance of tolerance, and mutations that impair this function may predispose individuals to intestinal disease. Future work will determine if the role of miR-10a in preventing Treg plasticity is unique to gut-expressed Tregs or broadly applicable to Tregs found at extra-intestinal sites.

Altogether, relatively few studies have examined gut-specific roles of well-known hematopoietic miRNAs, potentially due to assumption that their known functions in peripheral sites will carry over into the GI tract. While this may be true in some cases, it is possible that expression levels, targets, and the overall functions of miRNAs are distinct in the intestine versus extra-intestinal sites in the body. Furthermore, miRNAs that appear to play little or no roles in the systemic immune system may prove to have exclusive functions in unique populations of leukocytes within the gut. Because hematopoietic-derived immune cells interact heavily with the microbiota within the intestinal environment, understanding the roles of miRNAs within gut leukocytes will be key to understanding molecular pathways in which the host and microbiota interact, and this may differ from how a given miRNA functions in extra-intestinal sites.

## miRNAs IN NON-HEMATOPOIETIC-DERIVED GUT CELLS

Intestinal epithelial cells (IECs) are now recognized as non-hematopoietic cells that exhibit immunological functions, including the ability to directly recognize microbial products via PRRs (Vaishnava et al., 2008) and to secrete mucus (goblet cells) and antimicrobial peptides (paneth cells) in response to these microbes. As miRNAs regulate many immunological and developmental pathways, they may have significant functional relevance within IECs.

Generally, disruption of miRNA processing pathways in the intestinal epithelium results in susceptibility to intestinal infection. This was demonstrated through the specific deletion of *Dicer1* within IECs in mice (Dicer1^loxP/loxP^; Villin-Cre, referred to as Dicer1ΔIEC mice; McKenna et al., 2010; Biton et al., 2011). As Dicer is required for the processing of miRNAs into their mature form, its deletion abolishes miRNA function. These studies found that the deletion of Dicer from IECs results in mice with impaired growth, metabolism, and water retention. Additionally, Dicer1ΔIEC mice displayed a reduction in goblet cells, an increase in infiltrating inflammatory immune cells, and disorganized intestinal architecture. These mice also had increased intestinal permeability, decreased mucus production, and decreased Th2 cytokines and factors (Biton et al., 2011). Consequently, Dicer1ΔIEC mice were more susceptible to *Tricuris muris* infection and displayed IBD symptoms and inappropriate Th1 responses during infection. Finally, Biton et al. (2011) revealed that miR-375 was a critical IEC-expressed miRNA that was required for proper IEC differentiation and that it mediated communication with T cells to induce Th2 responses when appropriate.

One miRNA that appears to have similar targets in IECs as compared to other hematopoietic cell types is miR-146a. miR-146a directly targets essential TLR downstream signaling genes including TNF receptor associated factor 6 (TRAF6) and interleukin1 receptor-associated kinase 1 (IRAK1) to downregulate inflammation (Boldin et al., 2011; Zhao et al., 2011, 2013). In a mouse model of intestinal ischemia/reperfusion (I/R) injury, Chassin et al. (2012) showed that IEC expression of IRAK1 led to increased inflammation and tissue damage. When miR-146a was induced in IECs *in vitro*, expression of inflammatory chemokines and the effects of hypoxia were reduced. *In vivo*, miR-146a was directly injected into mice or its expression induced by DIM, and mice showed reduced disease and inflammation during I/R injury due to miR-146a targeting and downregulating expression of IRAK1 in IECs. Utilizing conditional knockout mice to examine the roles of IRAK1 and miR-146a specifically in IECs during this intestinal injury model, as well as in other intestinal disease models, would bring about further understanding of the cellular basis of miR-146a function in the gut.

microRNAs within non-hematopoietic cells may also a play a role in the development of and protection from intestinal cancers. The epithelium requires proper signaling mechanisms for normal development, differentiation, renewal, and repair of the intestinal tissue (Chivukula et al., 2014). Many of these signaling pathways require regulation by miRNAs, and colorectal cancer (CRC) can arise if dysregulated. One study found that miR-143 and miR-145, which are abnormally expressed during human CRC, are required for proper regeneration of IECs (Chivukula et al., 2014). When miRs 143 and 145 were deleted, mice were unable to undergo proper intestinal renewal and wound healing. The authors found that these miRNAs were expressed specifically within the intestinal mesenchyme and that deletion of miRs 143 and 145 from mesenchymal tissue resulted in a similar phenotype. Consistent with this, these miRNAs were found to target IGFBP5, an inhibitor of IGF signaling, which is required for regeneration of IECs. Thus, miRNAs expressed specifically within non-hematopoietic-derived mesenchymal cells are required to maintain intestinal regeneration and their dysregulation could result in improper growth and proliferation, potentially giving rise to intestinal cancers.

Another example, in which known miRNA functions are relevant in IECs, is the interplay of the RNA-binding protein LIN28 and the Let7 family of miRNAs. Let7 miRNA processing has been shown to be inhibited by LIN28 in models of cellular reprogramming, growth, and oncogenesis (Viswanathan and Daley, 2010). One group examined this interaction in the context of the intestine and found that overexpression of LIN28B in IECs led to abnormal intestinal architecture, crypt expansion, Paneth cell loss, and formation of intestinal tumors. (Madison et al., 2013). A similar phenotype was seen in miR-Let-7c2 and Let-7b deficient mice, indicating a disease-promoting role of LIN28B in targeting and degrading these miRNAs. Expression of Let-7 in the intestine reversed the hyperplasia and Paneth cell loss seen in the LIN28B overexpressing mice. This study indicates that Let-7 miRNAs are important for preventing overgrowth of IECs downstream of the oncogenic RNA-binding protein LIN28B. Expression or modulation of this miRNA family could either prevent or cause CRCs.

The ability of IECs to form tight junctions is important in creating an effective barrier between the host and commensal microbiota. Defective intestinal tight junction barriers are seen in IBD and other intestinal diseases and result in increased inflammation (Ye et al., 2011). Recently, miRNAs have been shown to play a role in regulating intestinal epithelial tight junction permeability. For example, Dicer1ΔIEC mice showed decreases in cells expressing the epithelial tight junction protein, claudin, and a weakened barrier (McKenna et al., 2010). In another study, stimulating IECs with TNFα* in vitro* led to increased expression of miR-122 (Ye et al., 2011). miR-122 directly targets occludin mRNA, which encodes a protein that forms intestinal tight junctions. Thus, expression of miR-122 increased intestinal epithelial permeability. Mice overexpressing miR-122 displayed decreased intestinal barrier function as a result of downregulated occludin expression. In this way, miR-122 may be a valid target in diseases involved in intestinal barrier and permeability, such as IBD.

Altogether, miRNAs play pivotal roles in IECs and other non-hematopoietic-derived intestinal cells, which can contribute to the protection of the host tissue from pathogenic invaders and disease. As common immunological pathways are essential within IECs, miRNAs that regulate these pathways also have relevance within these non-hematopoietic cells. For example, conditional deletion of the TLR adaptor protein MyD88 within IECs resulted in loss of barrier function and defective immunity from commensal bacteria (Vaishnava et al., 2011; Frantz et al., 2012). Thus, miRNAs that have known targets in immune signaling pathways downstream of MyD88 can also play important regulatory roles within IECs. Generally, based on the above studies and others, the expression and function of miRNAs within non-hematopoietic cells of the intestinal immune system play a large role in maintaining intestinal homeostasis.

## GUT MICROBIOTA AND miRNAs

In discussing the functions and features of the mammalian intestine, one cannot ignore the large contribution to host physiology by the gut microbiota. It is estimated that approximately one hundred trillion commensal organisms reside within the human GI tract (Phillips, 2009; Hooper and Macpherson, 2010). These microbes are extremely diverse in their taxonomy, communities, and functions. The gut microbiota communicates directly with the host via the production of metabolites, peptides, and other signaling molecules. MiRNA expression by the host may play a significant role in determining how microbiota-produced signals are received by the host, and miRNAs may balance the fine line between maintaining an effective barrier and preventing inappropriate inflammation in response to the microbiota.

It is known that the microbiota modulates expression of host genes, as expression profiles of WT germ-free (GF) mice are markedly different from WT specific pathogen free (SPF) mice (El Aidy et al., 2013). New studies are beginning to examine whether this communication between host and microbiota involves miRNAs. In one such study, GF mice were colonized with microbiota from SPF mice and their miRNA profile was examined via microarray (Dalmasso et al., 2011). Nine miRNAs were differentially expressed in the ileum and colon of SPF-colonized compared with uncolonized GF mice. This differential expression of miRNAs within the mouse gut was predicted to alter the expression of hundreds of miRNA target genes. For example, miR-665, which was downregulated in SPF-colonized mice compared with GF mice, targeted the Abcc3 gene (an ATP-binding cassette transporter) in the colon. In a similar study, the differences in miRNA expression within the cecum between GF and SPF mice were examined (Singh et al., 2012). Sixteen miRNAs were expressed differentially between GF and SPF mice. Upon analyzing networks of genes involved in intestinal barrier function that may be regulated by these miRNAs, the authors propose that the gut microbiota modulates host miRNA genes, which then target and regulate the intestinal barrier.

In another investigation, the role of the microbiota in regulating host miRNA expression was examined in the context of *Listeria monocytogenes* infection (Archambaud et al., 2013). GF mice had a greater *Listeria* burden when compared to infected SPF mice in multiple tissues. The authors hypothesized that these higher bacterial counts were due to the lack of microbiota, which prime host immune responses via changes in gene expression. They performed gene expression analysis of protein-coding and miRNA genes in SPF and GF infected and uninfected mice and observed that five miRNAs were downregulated in SPF mice during infection but not in GF mice. These included miR-378 and miR-200c. The downregulation of these miRNAs due to the presence of microbiota led to increased expression of several protein-coding genes that were predicted targets of these miRNAs. Using the set of differentially expressed target mRNA and their corresponding miRNAs, the authors revealed a miRNA-mRNA network in which microbiota-mediated miRNA expression primed the intestinal immune system to strengthen the barrier and combat *Listeria* infection. Additionally, the authors defined the ten miRNAs, including miR-143 and miR-215, that are most highly expressed within the ileum of SPF and GF mice before and during infection. These miRNAs may be considered ileal “signature” miRNAs.

While the above studies highlight potential roles for specific miRNAs in host-microbiota interactions, further investigation is necessary to reconcile differences between the studies and to find consensus miRNAs that may be more significantly involved in crosstalk between host gut tissue and the microbes that reside in this locale. So far, studies have examined miRNA expression profiles within varied intestinal sections; the microenvironments of these sections are largely different when compared to one another. Furthermore, these tissues contain a heterogeneous population of cells, which may not reveal miRNAs involved in host–microbial interactions within specific intestinal cell types. Utilizing cell sorting and RNA-sequencing to analyze gene expression may identify miRNAs that are differentially expressed within specific and/or rare gut cell types upon exposure to microbes. Additionally, further experimentation is necessary to understand cellular mechanisms and biological importance of the candidate miRNAs uncovered in these studies.

Some work has also been done to begin defining specific mechanisms by which miRNAs are induced by products of the microbiota. In one such investigation, expression of a subset of miRNAs was downregulated in human colon cells treated with butyrate, a beneficial short-chain fatty acid (SCFA) derived from commensal bacteria (Hu et al., 2011). These miRNAs, particularly those of the miR-106b family, were conversely expressed at high levels in colons of human colon cancer patients. Butyrate blocked expression of the miR-106b family to allow for increased expression of p21 in a miRNA-dependent manner. p21 is a cell cycle arrest protein important in preventing various cancers. Importantly, miR-106b reversed the anti-proliferative effects of butyrate via direct targeting of p21. Thus, a product of the microbiota regulates gene expression of host miRNAs to prevent colonic disease and cancer. The cellular mechanism by which butyrate directly modulates miRNA gene expression has yet to be investigated.

Another study demonstrated that miR-10a is highly expressed in the intestines and can be modulated by the gut microbiota through TLR signaling on dendritic cells, which suppresses expression of miR-10a (Xue et al., 2011). In experiments characterizing relevant targets and pathways, miR-10a directly targeted and downregulated expression of IL-12/23p40, an important cytokine for innate inflammatory responses in the GI tract. Mice with colitis had decreased expression of miR-10a and thus high levels of IL-12/23p40. In this instance, the microbiota signals to the host to downregulate expression of miR-10a, which targets a portion of host innate immunity. Another miRNA, miR-107, targeted a component of the IL-23 receptor, IL-23p19 (Xue et al., 2014). Like miR-10a, the expression of miR-107 was downregulated by the microbiota, as GF mice showed high levels of miR-107, which was decreased following microbial colonization. Mice with colitis displayed low levels of miR-107 and high levels of IL-23p19, indicating the importance of this miRNA in downregulating inflammation in response to commensals. Proper crosstalk between the gut microbiota and miR-107 in intestinal immune cells, which targets IL-23p19, can play a role in maintaining homeostasis.

Overall, signals from the microbiota have the ability to alter expression of miRNAs. In turn, miRNAs can target immune-related mRNAs that have the ability to impact responses to microbes and thus shape commensal communities. Thus, crosstalk between microbiota and miRNAs is required for shaping intestinal immune responses and maintaining homeostasis.

## miRNAs AND HUMAN INTESTINAL DISEASE

Many human diseases are related to dysregulation of the intestinal immune system and of the microbiota, including allergies, various autoimmune diseases, and cancers (Round and Mazmanian, 2009; Kamada et al., 2013; Sears and Garrett, 2014). One of the most prominent and well-studied class of intestinal disease is inflammatory bowel disease (IBD). IBD is caused by chronic and inappropriate inflammation within the gastrointestinal tract, and this affects approximately 1.4 million Americans. Furthermore, CRC risk is significantly increased in patients that have IBD (Neurath, 2014). Together, this has placed a considerable burden on the healthcare system (Abraham and Cho, 2009). The most common types of IBD are Crohn’s Disease (CD) and ulcerative colitis (UC), and each of these display different causes and pathologies. Although genetic and environmental factors are known to play a role in the etiology of IBD subtypes, the specific factors that directly cause these diseases are not fully understood. Furthermore, CD, UC, and other intestinal diseases appear to display various pathologies, outcomes, and treatment options depending on the individual (Dalal and Kwon, 2010), emphasizing the need for more diagnostic and therapeutic reagents in the clinic.

Recently, miRNA expression profiles have been shown to change in people suffering from IBD. Patients with UC and CD have distinct miRNA profiles that are unique to their disease stage when compared with healthy controls. This is true for both intestinal tissue biopsies and peripheral blood (Dalal and Kwon, 2010; Wu et al., 2011; Pekow and Kwon, 2012; Iborra et al., 2013; Lin et al., 2014). Among the miRNAs found to have altered expression during UC and CD were miR-16 (Wu et al., 2010), miR-146a (Lin et al., 2014), miR-31 (Lin et al., 2014), miR-340^∗^ (Wu et al., 2011), and miR-199a-5p (Wu et al., 2011). Some of these miRNA expression profile studies during IBD have been summarized in two recent reviews (Dalal and Kwon, 2010; Pekow and Kwon, 2012). It is important to note that each study has sampled a different group of patients, disease types and states, as well as tissue types. This likely explains why there does not appear to be a common group of miRNAs that are associated with IBD across all studies. Future work must be done to reconcile the differences in miRNA profiles in each study and to clarify which miRNAs are common or unique to distinct disease types. These miRNA profiles might be used to accurately diagnose IBD types and predict patient prognoses. Additionally, the concept of utilizing blood miRNAs as biomarkers for intestinal disease is promising because collection would be non-invasive. Even so, further experimentation needs to be done to understand the functional significance of altered expression of specific miRNAs in various human tissues and cell types during disease.

The roles of some miRNAs and their targets during IBD and intestinal disease are beginning to be elucidated. For example, during active pediatric UC, levels of phosphorylated Stat3 were increased in colonocytes, and correlated with low expression of miR-124 in diseased patients (Koukos et al., 2013). Furthermore, miR-124 directly bound to and downregulated expression of Stat3. High levels of Stat3 and corresponding low levels of miR-124 were only seen in pediatric patients with active UC and not in adults, pediatric CD, pediatric inactive UC, nor healthy patients. This was also observed in mouse models of colitis. Dysregulation of the Stat3 pathway occurs in diseased patients due to hypermethylation of miR-124. This study implicates miR-124 and the Stat3 pathway as highly relevant potential therapeutic targets for treatment of pediatric UC. Even so, whether downregulation of miR-124 via hypermethylation is a cause or effect of IBD in children has not yet been elucidated. In another study, Brain et al. (2013) showed that human CD patients with polymorphisms in the NOD2 gene were unable to upregulate expression of miR-29. In this way, miR-29 could not repress downstream cytokine signaling pathways that were attributed to worsened disease. In general, these findings support the validity of miRNAs and their experimentally verified targets as important players in human IBD.

Single nucleotide polymorphisms (SNPs) in miRNA genes may also play a role in susceptibility to intestinal diseases (Pekow and Kwon, 2012; Gazouli et al., 2013). SNPs within miRNAs could alter their expression, processing, and functional targeting. Thus, the presence of certain miRNA SNP variants in humans can be associated with and may even be functionally relevant (Jin and Lee, 2013). The rs2910164 SNP within the miR-146a gene on chromosome 5 has been shown to decrease mature miR-146a levels and subsequently increase its target genes (Jazdzewski et al., 2008; Shao et al., 2014). This miRNA SNP has been implicated in numerous human diseases, including various cancers (He et al., 2012), papillary thyroid carcinoma (Jazdzewski et al., 2008), and sepsis (Shao et al., 2014). Even so, a general consensus within studies and meta-analyses regarding disease associations with rs2910164 has not been reached, as group size, methods of statistical analysis, and the ethnic groups sampled can affect the conclusions made in each study. In relation to the GI tract, this miR-146a SNP was associated with CD (Gazouli et al., 2013), while no significant association was observed with UC patients (Okubo et al., 2011; Gazouli et al., 2013). Patients with this SNP variant were also found to have increased risk of intestinal metaplasia and dysplasia during *H. pylori* infection (Song et al., 2013). Furthermore, this polymorphism in miR-146a predicted susceptibility to CRC and disease-specific survival outcome (Chae et al., 2013; Ma et al., 2013). MiR-146a downregulates inflammation by targeting components of the NFκB pathway (Boldin et al., 2011; Zhao et al., 2011, 2013), and these studies suggest that a change in one nucleotide within this miRNA could alter the intestinal inflammatory state via dysregulation of NFκB and other related pathways. However, the functional consequences of this polymorphism remain incompletely understood. Taken together, these findings provide evidence that the altered function of immune-related miRNA genes can influence the susceptbility to, and outcome during, intestinal diseases.

As IBD patients carry an increased risk of developing CRC, miRNAs also have potential as biomarkers, prognostic tools, and therapeutic targets during cancers of the GI tract. While multiple factors may influence CRC, miRNAs have been shown to play important roles in the molecular pathways that can give rise to intestinal cancers. These include pathways involved in inflammation, chromatin formation, stem cell signaling, apoptosis, and others (Liu and Chen, 2010; Chivukula et al., 2014). Expression profiling has indicated that miR-31, miR-21, and miR-191 are upregulated (Liu and Chen, 2010), while miR-143, miR-145, and miR-451 are downregulated in CRC tumors (Liu and Chen, 2010; Chivukula et al., 2014). Another study, using an RNA-sequencing approach, found that miR-10a-5p, miR-21-5p, miR-22-3p, miR-143-3p, and miR-192-5p are among the most abundantly expressed miRNAs in a CRC cohort (Schee et al., 2013). A pathway analysis revealed that these altered RNAs could affect many cellular signaling pathways including Wnt, MAPK, and TGFβ, all of which are linked to oncogenesis. Animal studies utilizing genetic manipulation of miRNAs within the intestines are currently being carried out to understand cellular mechanisms of CRC-related miRNAs that have been found in human studies, and a recent report has found critical roles for miRNAs 143 and 145 in pathways that can lead to CRC (Chivukula et al., 2014). Although much work remains, it has become clear that miRNAs within cells of the intestines are relevant during human IBD, CRC, and other intestinal diseases that stem from perturbations in intestinal homeostasis. This underscores the importance of miRNAs in maintaining intestinal homeostasis in humans.

## FUTURE DIRECTIONS AND CONCLUDING REMARKS

In general, these investigations suggest that miRNAs are likely indispensable regulators of host-commensal interactions that are required for proper intestinal homeostasis. Because miRNAs fine-tune targets by downregulating their expression 1.2–4-fold (O’Connell et al., 2012), they prevent imbalances that eventually lead to loss of homeostasis and disease. miRNAs may also prove to be effective drug targets in terms of treating intestinal diseases. Extensive future studies are required to further define specific mechanisms of miRNA function in intestinal immunity. This will unveil how miRNAs influence the balance between maintaining beneficial resident microbes and eliminating those that are harmful within the gut.

Tying together emerging concepts in the miRNA field with those in the GI tract and microbiota fields will bring about a comprehensive understanding of cellular processes that control intestinal homeostasis. One of these novel concepts is the transfer of miRNAs between cells via exosomes (Stoorvogel, 2012). In the future, it will be important to determine if this mode of exosomal miRNA transfer occurs and has biological relevance within the intestines to modify gut homeostasis and commensal populations. Another emerging concept regarding the microbiota involves the important role of gut commensals in contributing to extra-intestinal effects. For example, recent studies have determined that the gut microbiota can impact the biology of extra-intestinal sites, such as the brain, heart, and liver and as a consequence can influence diseases including MS, autism, cardiovascular disease, and obesity (Wang et al., 2011; Collins et al., 2012; Hsiao et al., 2013; Kamada et al., 2013; Zhao, 2013); miRNAs may play important roles in the communication process between host and microbiota at extra-intestinal sites. miRNAs may also function in cells as they respond to gut microbial metabolites and other products. Commensal-derived metabolites, such as short-chain fatty acids (SCFAs) and bile acids, play important roles in development and function of immune cells within the gut and can contribute to immunological and metabolic phenotypes within the host (Brestoff and Artis, 2013).

miRNAs and host-commensal interactions have been independently linked to human development, health, and disease in the past. Now, as we begin to explore how these systems are integrated, a greater understanding of how host-commensal interactions are regulated will undoubtedly emerge. This will provide novel therapeutic insights that will help combat the wide range of diseases that are related to the gut.

## Conflict of Interest Statement

The authors declare that the research was conducted in the absence of any commercial or financial relationships that could be construed as a potential conflict of interest.
